# New and Old Code of Ethics in the State of New York

**Published:** 1884-03

**Authors:** 


### NEW AND OLD CODE OF ETHICS IN THE STATE OF NEW YORK.

At the annual meeting of the Medical Society of the State of New York, held at Albany the 5th, 6th and 7th of February, the contest over the old and new Code was renewed, and, after the usual speech-making upon such occasions, the vote was taken and resulted in the defeat of the old Code faction by a vote of 105 for old Code and 124 for the new Code. So ends the contest, so far as the Medical Society of the State of New York is concerned, but the end is not yet. It was stated upon the floor by the President, Dr. Alex. Hutchins, that there was a large majority of the physicans of the State who favored the old Code, or the Code of the American Medical Association, but they were not there to vote. This whole contest has been unnecessary and bitter, rupturing long existing friendships and pleasant associations in many localities in the State of New York, without any good to show for it—without any advance in science, or profit to the profession.

The truth is, the new Code faction commenced the fight at the wrong time and place. They should have presented their grievances to the National Medical Association, to which all bore allegiances, and there demanded a repair of the wrong, if there was a wrong, and there to have made the fight for the right, if they were right. But they chose to secede without notice, abolish the laws of the National Association, which up to that time were the laws of their own society—made laws to suit themselves, and then sent delegates to the American Medical Association for admittance, as much as to say, we have abolished your laws in our association—have made "laws unto ourselves"—what are you going to do about it? The National Association, by this course, was driven to one of two alternatives—either to admit their delegates, and become subordinate to the Medical Society of the State of New York, thus yielding her prestage as the legislative body and law-makers to the profession of the United States, or to refuse their delegates admission to seats in their body. Without any hesitancy she did the latter—refusing their delegates admission.

The American Medical Association has ever been ready, not only to listen to the grievances of any respectable number of its members, but to have them discussed and acted upon. Only a few years ago the Association ordered a circular sent to every member of the Association, asking the questions (can't give language) something like the following: Are you in favor of the Code of Medical Ethics as it now stands? If you prefer a change, in what particular? giving article, section, etc. In reply to this circular, there was an overwhelming majority for the Code of Ethics to remain as it was.

We are more confirmed every day in our opinions as to the prime cause of the agitation of this subject, and the object hoped to be attained by some of the leaders of this movement, but at present we are not disposed to discuss this feature of the subject. This agitation has resulted, as many predicted in 1882, in the organization of a new medical society in the State of New York, and from the New York journals we learn it is an accomplished fact. The organizaion is to be known as the "New York State Medical Association" and was organized in Albany, on the 6th of February, by sixty-five members of, (I suppose, seceding members) from the Medical Society of the State of New-York. They adopted the old or national Code of Ethics. Dr. H. D. Didama, of Syracuse, was elected President; Dr. E. D. Furguson, of Troy, Secretary; and Dr. J. H. Huston, of New York, Treasurer. A council of sixteen members were elected. The first annual session will be held on the 18th November, 1884, in New York city.

